# Supplementary material for: AOX Affects the Synthesis of Polysaccharides by Regulating the Reactive Oxygen Species in Ganoderma lucidum
Source: Foods. 2025 Feb 27;14(5):826. doi: 10.3390/foods14050826 (PMC11898911; doi:10.3390/foods14050826)
Supplement: Supplementary file 1 [file foods-14-00826-s001.zip › foods-3424789-supplementary.pdf]

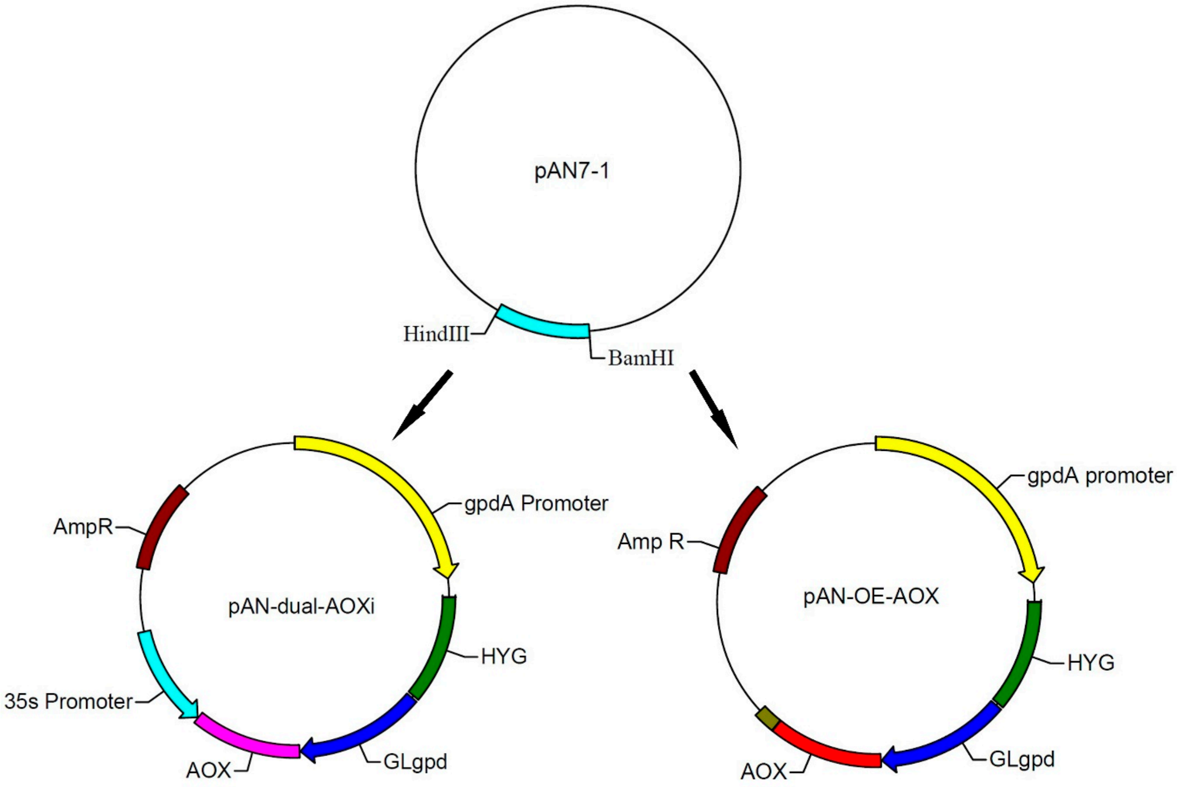

Figure. S1 Construction of silencing Vector and overexpression Vectors for G. lucidum *AOX*

In the silencing vector, the 35s promoter drives the antisense chain of *AOX* in the plasmid, and the glyceraldehyde-3-phosphate dehydrogenase promoter drives the sense chain of *AOX* gene.

Table S1 Oligonucleotide primers used.

Primer	Sequence (5′ to 3′)	Description
AOXi-F	ccctctcaacGCGGCCGCATGTCCTCATCCACGAGACATGA	Clone GLAOX gene
AOXi-R	taggcagctttGATATCTCAGTTCGCCTCCCCGCG	
gpd-F	TAGATGCCGACCGCGGATCCTCCAAAGCCGCTCTCATGG	Clone GLgpd gene
gpd-R	ATGAGGACATGCGGCCGCGTTGAGAGGGGGATGAAGAGTG	
GAPDH-F	TCGGCGGATGCTCCCATGTT	Housekeeping gene
GAPDH-R	GTGGTGCAGGAAGCGTTGGA	
RT-PMI-F	CTCGTCTTTGAAGCGGTCCAC	Detect the PMI expression
RT-PMI-R	CATACTTCCCCATCGCGTCT	
RT-PGM-F	CCGAAACCGTCCAGACCATCCT	Detect the PGM expression
RT-PGM-R	CCACCATCCGCCTTGTACTTGC	
RT-AOX-F	GTGGTGCAGGAAGCGTTGGA	Detect the AOX expression
RT-AOX-R	GCGAGGCTGTGGTTCACGAA	
RT-CAT1-F	CGTGTGCGTGGTGCCAAGTT	Detect the CAT1expression
RT-CAT1-R	AAGGAGTAGGCGGAGACGAGGT	
RT-CAT2-F	GGAGATCGAGCAGGTTGCGTTC	Detect the CAT2 expression
RT-CAT2-R	AAGACGGTGGCGGTGAGTGT	
RT-SOD1-F	TCATCGCCGTCTTCGTCGTTTG	Detect the SOD1expression
RT-SOD1-R	AGAGGGTTTCGCCGTTGAGGA	
RT-SOD2-F	TCACCGCGACGCGATTACGA	Detect the SOD2 expression
RT-SOD2-R	TCCTTGATGGTGCCGCCGTT	
RT-SOD4-F	GCCACATCAACCACTCGCTCTT	Detect the SOD4 expression
RT-SOD4-R	AGTCCGCCTCGATCTTCTGCTT	
RT-APX-F	AACTGGCGGCAGCAACTACG	Detect the APX expression
RT-APX-R	CAAGCGTCCAGAGGTCACCGTA	
RT-GPX-R	CAACGGCGACAACACGAACGA	Detect the GPX expression
RT-GPX-R	CGGTGAACGACCTTGCCTTCTT	
